# High resolution nanoscale chemical analysis of bitumen surface microstructures

**DOI:** 10.1038/s41598-021-92835-3

**Published:** 2021-06-30

**Authors:** Ayse N. Koyun, Julia Zakel, Sven Kayser, Hartmut Stadler, Frank N. Keutsch, Hinrich Grothe

**Affiliations:** 1 Christian Doppler Laboratory for Chemo-Mechanical Analysis of Bituminous Materials, Institute of Materials Chemistry, TU Wien, Getreidemarkt 9/BC, 1060 Vienna, Austria; 2 IONTOF GmbH, Heisenbergstrasse 15, 48149 Münster, Germany; 3 Bruker Nano-Surfaces Division, Östliche Rheinbrückenstrasse 49, 76187 Karlsruhe, Germany; 4 John A. Paulson School of Engineering and Applied Sciences, Harvard University, Cambridge, MA 02138 USA

**Keywords:** Nanoscale materials, Soft materials

## Abstract

Surface microstructures of bitumen are key sites in atmospheric photo-oxidation leading to changes in the mechanical properties and finally resulting in cracking and rutting of the material. Investigations at the nanoscale remain challenging. Conventional combination of optical microscopy and spectroscopy cannot resolve the submicrostructures due to the Abbe restriction. For the first time, we report here respective surface domains, namely catana, peri and para phases, correlated to distinct molecules using combinations of atomic force microscopy with infrared spectroscopy and with correlative time of flight—secondary ion mass spectrometry. Chemical heterogeneities on the surface lead to selective oxidation due to their varying susceptibility to photo-oxidation. It was found, that highly oxidized compounds, are preferentially situated in the para phase, which are mainly asphaltenes, emphasising their high oxidizability. This is an impressive example how chemical visualization allows elucidation of the submicrostructures and explains their response to reactive oxygen species from the atmosphere.

## Introduction

Bitumen is an important industrial product of mineral oil refining and is mostly used for the production of asphalt concrete. Its other main applications are waterproofing materials for asphalt roofing board and insulation purposes. Understanding the oxidation behaviour of this economically important material paves new ways to prevent atmospheric ageing and therefore can make a difference of many years in its lifespan and it saves energy and material resources^1–5^.

Surface composition often plays a major role in atmospheric ageing processes and, as with many materials, surface chemistry is different from the bulk and therefore should be given full consideration when investigating oxidation behaviour. Understanding the surface chemical heterogeneity is of paramount importance in recognizing the surface properties and therefore the interaction of asphalt with the atmosphere. The degree of atmospheric oxidation depends on the surface material properties and its chemical make-up. Traditional approaches to study surface microstructure composition generally lack the resolution and sensitivity to characterize individual domains of the surface and therefore resolving its chemical heterogeneity is challenging. Bitumen is a complex viscoelastic organic material which consists of over thousands of different types of chemical compounds mainly hydrocarbons with functional groups containing heteroatoms such as nitrogen, oxygen and sulfur^6–8^. To reduce the degree of complexity of bitumen chemistry and study bitumen components, a separation of bitumen via polarity-chromatography into saturates, aromatics, resins and asphaltene (SARA) fractions has widely been accepted^8^. Unfortunately, a direct correlation between SARA fractions and microstructure is a missing link, since the formation of the microstructures is thought to be the result of the differences of the polarities of the molecules and their molecular interactions^9^. Earlier studies focusing on the surface microstructures of bitumen have revealed that the presence and appearance of surface microstructures varies considerably depending on crude oil source, oxidation status, thermal history and sample preparation method^10–14^.

**Figure 1:**
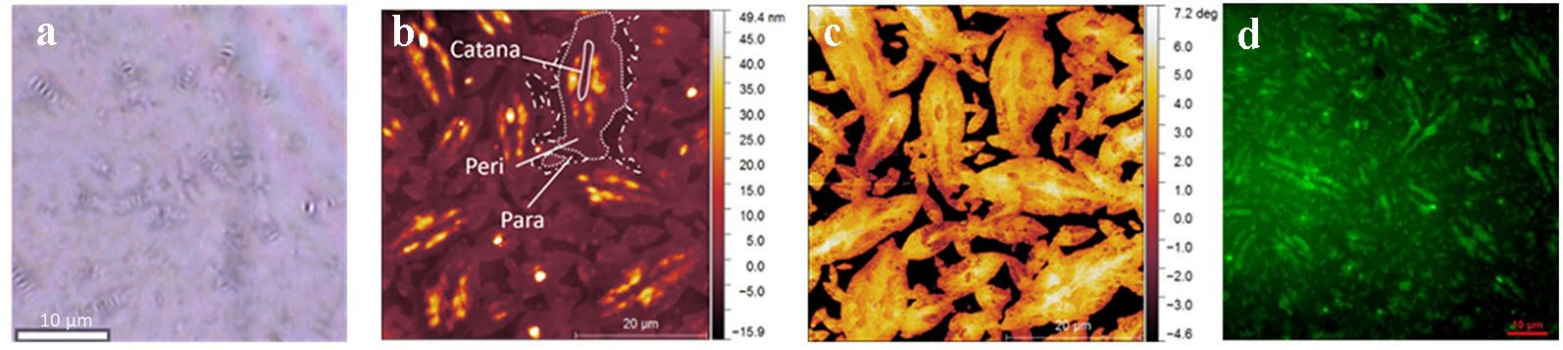
Bitumen surface demonstrated with various microscopic techniques. (**a**) Light microscopy, (**b**) AFM (topography image), (**c**) AFM (phase image) highlighting catana, peri and para phase and (**d**) fluorescence microscopy showing brightly fluorescing phases.

Previous studies focusing on the addition of wax to bitumen show extensive wax segregation with a distinct circular shape on the surface as well as saturates enriched ‘bee-like’ structures previously observed with atomic force microscopy (AFM), environmental scanning electron microscopy (ESEM) and chemical analysis using time of flight–secondary ion mass spectrometry (ToF–SIMS)^15–21^. For untreated bitumen distinct domains namely the catana, peri and para phase occur^19^. It has also been found that the natural wax content plays a crucial role in the shape and size of the bee-like structures^21–24^. The first AFM studies show a heterogeneous composition on bitumen surface with a ‘*bee-like*’ structure^19,25,26^. The impression ‘*bee-like*’ structure stems from the AFM topography images (with the traditional color scale black-orange-yellow-white) of bitumen surface, where the top view shows striped microstructures that resemble bumble-bees. The alternating higher and lower topography was eponymous for the impression of bees, surrounded by a flat area. The three different domains were named *catana*, *peri* and *para* phase (Fig. 1b). *Catana*, or *catanic* phase, was named according to the Greek terms *cata* (high to low) and *ana* (low to high), also attributed as the ‘*bee-like*’ structure. The area surrounding the Catana is called *peri* (around in Greek) phase, which is next to *para* (neighbouring) phase^27^. Over the years the surface phases have achieved significance due to their differences in chemical composition leading to preferential oxidation phenomena.

In the past, the structural impact of asphaltene molecules has been unravelled with Atomic Force Microscopy (AFM) and Scanning Tunnelling Microscopy (STM)^28^ and molecular agglomeration has been compared to modelling studies^29–31^. High bulk resolution of bitumen has been achieved with high-field Fourier-Transform Ion Cyclotron Resonance (FT-ICR) mass spectrometry (MS)^32^. Also, several other spectroscopic and microscopic methods for unravelling bitumen surface microstructures were developed and applied. Atomic Force Microscopy^11,19,33^, Fluorescence Microscopy^14,34–36^, AFM-infrared (AFM-IR)^37^, Time-of-Flight Secondary Ion Mass Spectrometry (ToF–SIMS)^15,16,18,20^ studies have shown the morphological and chemical heterogeneity of the surface. However, fluorescence microscopy, AFM-IR and ToF–SIMS provide only partial information about the surface chemistry and a correlation between various spatial chemical characterization methods providing different chemical information with increased lateral resolution is needed. These studies succeeded in portraying the heterogeneity of the surface. Nevertheless, achieving high resolution spectral information of the microstructures and a comprehensive study with various complementary techniques is missing but is vital to draw conclusions about the chemical composition of the surface microstructures. This will allow to lay the foundation required to perform atmospheric oxidation studies, with important questions regarding preferential oxidation mechanisms on specific surface areas. Given the chemical and morphological heterogeneity of the surface the compositional information may explain where and how oxidation starts and progresses.

A complete characterization of surface phases is only possible with the combination of different techniques: (a) Fluorescence microscopy studies have shown fluorescent centres which were attributed to aromatics and resin fractions^34^. (b) AFM-IR observed the same centres and recorded sulfoxide stretching mode of organic sulfate esters at 1080 cm^−1^ and carbonyl stretching of ketones at 1700 cm^−1^ for the para-phase^38^. (c) ToF–SIMS measurements found chemical heterogeneities on the surface, however, the typical microstructural features occurring on bitumen surface, catana, peri and para phase, could not be resolved by means of the main molecular components aromatics, saturates, resins and asphaltenes^15^.

The added value of this study is to combine techniques a, b and c and to gain chemical information on the main phases of the surface microstructures to unravel the puzzle of bitumen microstructure and simultaneously learn where oxygenated compounds are incorporated and how possible chemical ageing mechanisms proceed on the bitumen surface which finally result into macroscopic potholes.

## Results

### Incident light microscopy, fluorescence microscopy and AFM imaging

Incident light microscopy, fluorescence microscopy and AFM topographical and phase imaging reveal typical bitumen surface microstructures. Surface morphology is imaged and identified with conventional incident light microscopy (Fig. 1a) and atomic force microscopy (Fig. 1b). Typical bitumen surface microstructures as referred to as ´bee-structures´ with sharp and distinctive shapes with catana, peri and para phase known from the literature^27,39^ and they are present on the surface and illustrated in Fig. 1b. The correlated AFM Phase image (Fig. 1c) reveals typical surface phases with strong phase contrast indicating the differences in the morphology and mechanical properties of the microstructure such as hardness and elasticity which are mainly correlated to chemical differences. Fluorescing elongated catana and peri phase regions of the microstructure together with the non-fluorescent domain (Fig. 1d), are a clear indication of chemical heterogeneity along the surface microstructure. With asphaltenes not being capable of fluorescence in the selected excitation and emission range and saturates being non-fluorescent due to their chemical nature, aromatics and resins are the main candidates for the catana and peri phase^34^.

### AFM-IR

**Figure 2:**
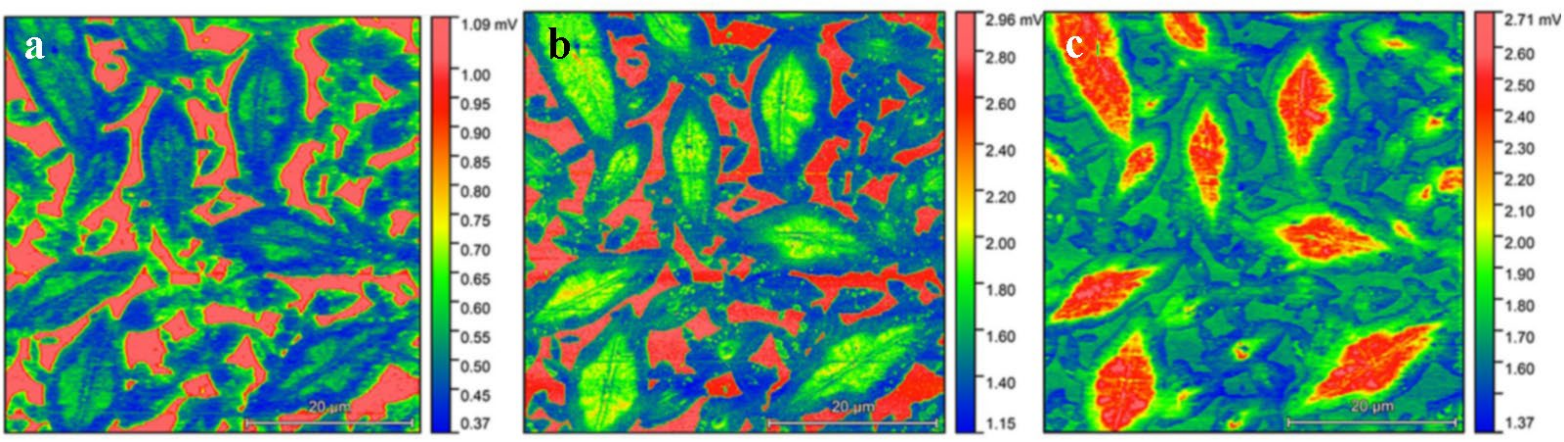
AFM-IR images at fixed wavelengths. AFM-IR mapping at (**a**) 1080 cm^−1^ (**b**) 1262 cm^−1^ and (**c**) 1376 cm^−1^. Here, red, green and blue areas indicate high, medium and low concentrations, respectively. Field of view 50 × 50 µm^2^.

**Figure 3:**
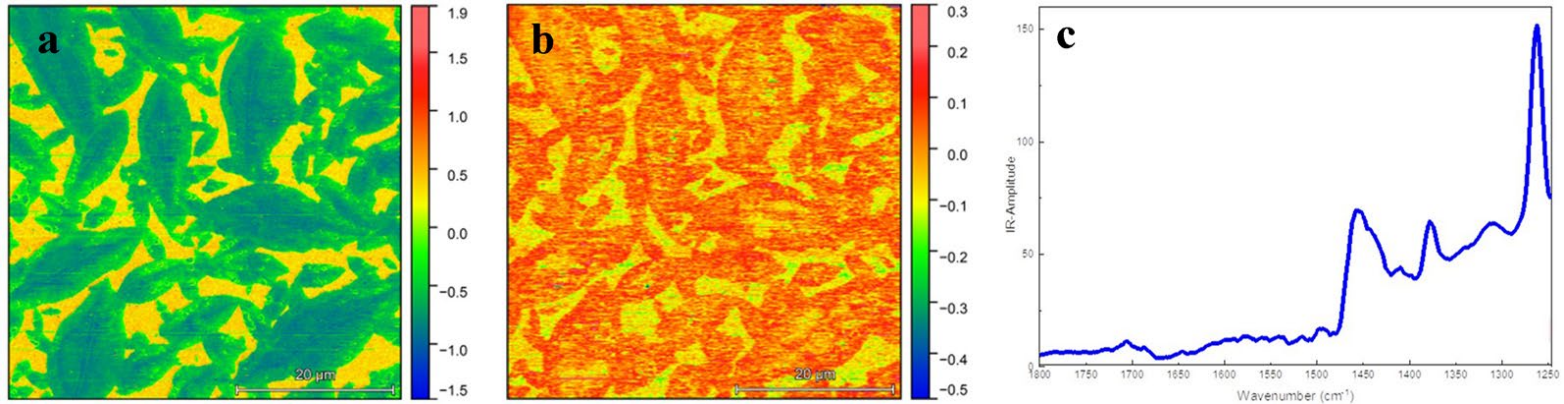
AFM-IR images at fixed wavelengths. AFM-IR mappings demonstrating (**a**) ratio I_1262_:I_1292_ (Aryl C–O str; peak 1262 cm^−1^ vs baseline 1292 cm^−1^), (**b**) ratio I_1378_:I_1400_ (aliphatic region; peak 1378 cm^−1^ vs baseline 1400 cm^−1^) and (**c**) representative AFM-IR spectrum.

Characteristic vibrations of the surface molecules (50 × 50 μm^2^ and a 5 × 5 μm^2^ field of view) were acquired by AFM-IR (at 10 nm lateral resolution). The focus of the AFM-IR measurements is on the functional groups that exhibit stark contrast in absorption features over the surface. Based on the changes of the absorption bands the functional groups on the surface exhibit major variations for sulfoxide functional groups ν(S=O) from organic sulfate esters at 1080 cm^−1^, ν(C_aryl_–O) from carboxylic acid ester at 1262 cm^−1^ and the characteristic methyl group δ_s_(CH_3_) at 1376 cm^−1^. IR maps at 1080 cm^−1^, 1262 cm^−1^ and 1376 cm^−1^ reveal that the functional groups are subject to significant changes and are directly attributed to the surface domains as shown in Figs. 2 and 3. Functional groups are present along the entire surface; however, the intensity is distributed unevenly. A variable distribution of the organic sulfate esters, carboxylic acid esters and aliphatic concentration is present rather than a sharp separation of the catana, peri and para phases.

The prominent band around 1080 cm^−1^ which is associated with the stretching vibration of sulfoxide groups ν(S=O) of organic sulfate esters, exhibits relatively higher concentration in the para phase, whereas the catana and peri phase exhibited slightly less absorption. As observed for different types of bitumen sulfoxide groups typically increase during bitumen oxidation^40–42^. Interestingly, the peri phase itself shows pronounced concentration differences within the peri area. The image recorded with the laser wavenumber fixed at the 1262 cm^−1^ ν(C_aryl_–O) IR absorption band shows a similar overall trend as the image of the ν(S=O) band but with a slightly higher absorption in the catana phase. The intensity of the ν(C_aryl_–O) band is higher in the para phase compared with the centre and the inner part of the peri phase. The lowest absorption of ν(C_aryl_–O) band was detected on the outer edge of the microstructures. Both Fig. 2a,b show accumulation of molecules containing S=O and C_aryl_–O groups in the para phase. The image visualizing the 1376 cm^−1^ characteristic modes for δ_s_(CH_3_) methyl group of the aliphatic hydrocarbons (CH_3_ deformation of aliphatic branches) exhibits the highest absorption in the centre of the microstructure mainly the catana and the inner peri phase and is lower at the outer edge of the para phase suggesting that aliphatics are mainly located in the catana phase. Interestingly, the matrix (para-phase) shows a higher intensity than the outer edge of the microstructures.

To better highlight the ν(C_aryl_–O) absorption and the aliphatic region in respect to the baseline, the ratio of 1262 cm^−1^/1292 cm^−1^ and 1378 cm^−1^/1400 cm^−1^ absorption images are visualized in Fig. 3a,b. AFM-IR ratio images reflect the chemical contrast more than AFM-IR single wavelength images and confirm that AFM-IR images in Fig. 2 are artefact free.

**Figure 4:**
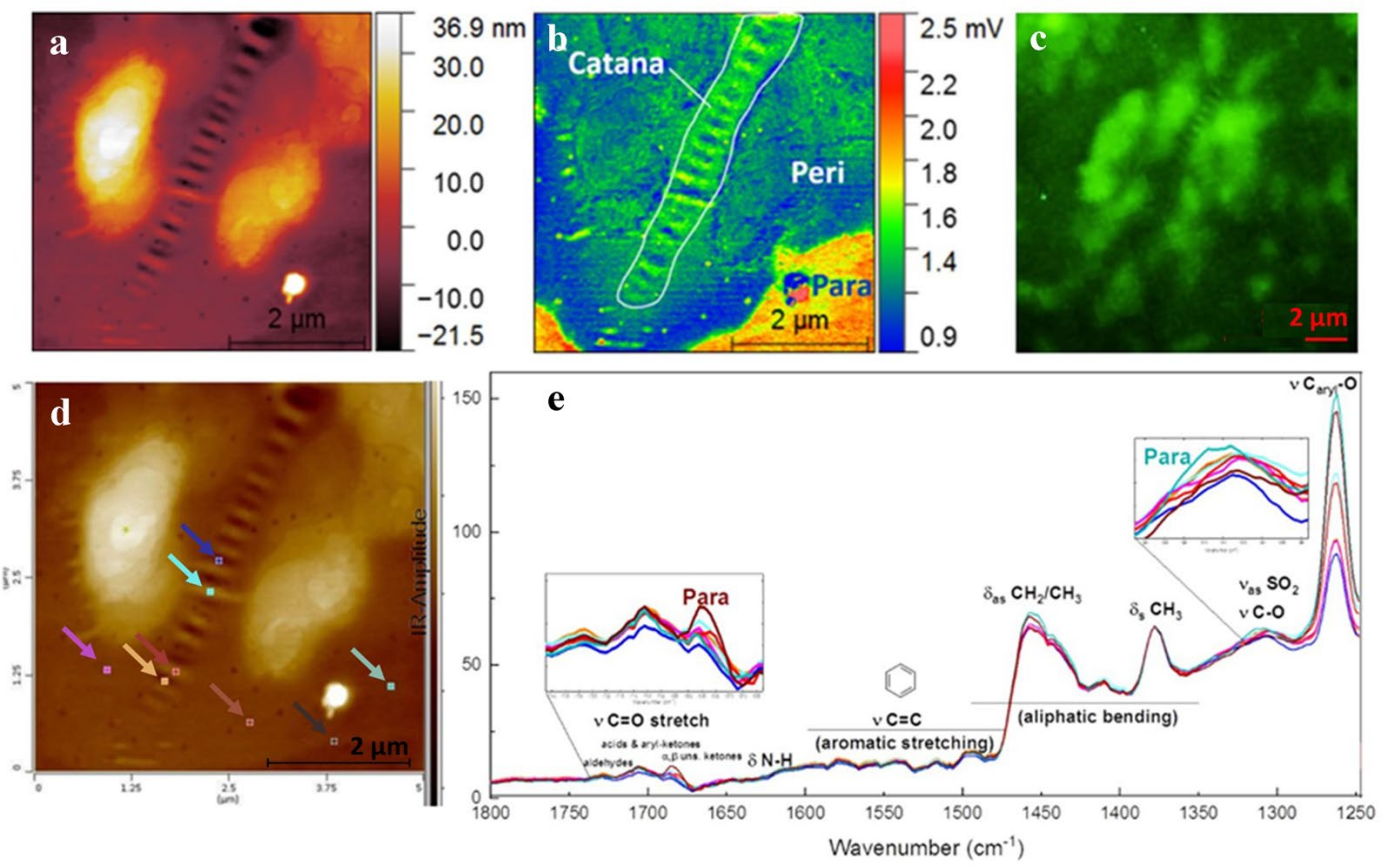
Single surface microstructure visualized with spectroscopic and microscopic techniques. (**a**) AFM topographic image of a single microstructure with respective (**b**) AFM-IR image at fixed wavelength 1262 cm^−1^ (**c**) corresponding fluorescence image of a single microstructure, exhibiting strong fluorescence on catana and peri phase, (**d**) AFM Image with colour-marked areas and their € localized AFM-IR spectra with the same colour scheme.

AFM-IR spectra, as shown in Fig. 4e, exhibit characteristic absorption bands for this type of bitumen: The absorption bands in the region 1750–1670 cm^−1^ centred at (i) 1729, (ii) 1706, and (iii) 1685 cm^−1^ can be attributed to carbonyl stretching vibrations ν(C=O) in (i) aldehydes^43,44^ (ii) carboxylic acids, aryl-ketones and esters^44,45^ and (iii) α, β—unsaturated/aromatic ketones, which show a higher absorption in the para-phase (Fig. 4d,e). Aromatic ring ν(C=C) stretching bands in the region 1625–1430 cm^−1^ (with the strongest bands at 1492 cm^−1^, 1515 cm^−1^, 1542 cm^−1^, 1560 cm^−1^, 1580 cm^−1^) occur at variable intensities. Series of carbonyl absorption bands which are similar to those of polycyclic quinones^46^: p-naphthoquinone (1664 cm^−1^), pyranthrone (1655 cm^−1^), helianthrone (1646 cm^−1^), dibenzanthrone (1638 cm^−1^), diphenoquinone (1626 cm^−1^) are observed. Due to the complicated nature of the absorption bands in the 1650–1430 cm^−1^ region and their changes during oxidation, an overlap of oxygenated and aromatic carbon groups is observed. The broad absorbance bands centred at 1630 and 1610 cm^−1^ correspond to aromatic ν(C=C) stretching vibration. Aliphatic absorption features centred at 1450 and 1375 cm^−1^ are attributed to symmetric and asymmetric bending modes of methyl and methylene vibrations. The strong absorption bands at 1410 and 1610 cm^−1^ are likely due to the symmetric and antisymmetric stretching vibrations of the carboxylate groups RCOO^−^. Amide δ_N–H_ bending vibrations are observed between 1650 and 1626 cm^−1^. Overlapping broad absorption bands are observed at 1308 and 1317 cm^−1^ indicating the presence of dialkyl sulfones CH_3_SO_2_ (asymmetric stretching vibrations ν_as_(SO_2_), ν(C–N) and carbon–oxygen ν(C–O) single bond stretching vibrations^47^.

Detailed chemical images of a single surface structure have been examined via AFM-IR at a high lateral resolution (10 nm). Distinctive catana structures with typical alternating ridges and valleys (see Fig. 4b Catana), which could previously only be portrayed with atomic force microscopy (see Fig. 4a), have been observed for the first time with this technique. Various spots on the surface were irradiated and compared to each other. The IR spectra corresponding to the marked spots in Fig. 4d are shown in Fig. 4e. Along the microstructure a sharp drop of the absorption band at 1262 cm^−1^ ν(C_aryl_–O) is observed with the strongest absorption in the para phase. The bending band at 1456 cm^−1^ attributed to aliphatic methylene band changes noticeably. It exhibits a relatively strong absorption at the outer part of the peri phase. The absorption band centred at 1310 cm^−1^ which is associated with ν_as_(SO_2_) and ν(C–O) exhibits a relatively high absorption in the para phase. α, β—unsaturated ketones exhibit the highest absorption in the para phase clearly indicating the accumulation of oxygenated compounds in the matrix (para phase). Fluorescence Microscopy (see Fig. 4c) captured the aromatic and resin compounds. The catana and peri phase exhibit strong fluorescence, while the para phase and the valley of the catana phase remained relatively low in fluorescence.

### AFM-ToF–SIMS

**Figure 5:**
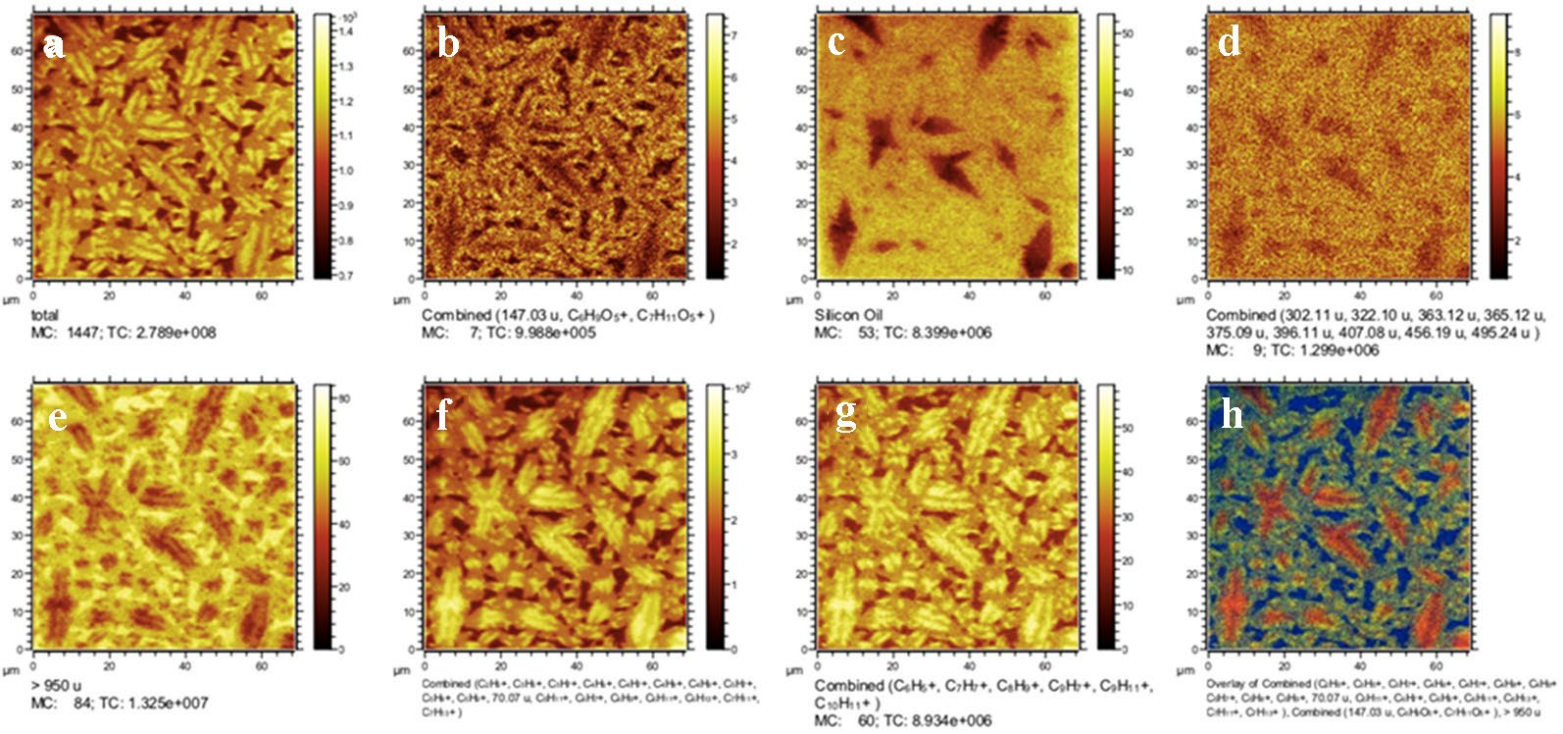
Polarity positive ToF–SIMS images of the bitumen surface highlighting various phases and their corresponding mass and suggested compound. Field of view: 70 × 70 µm^2^. Images created using SurfaceLab7 software. https://www.iontof.com/m6-tof-sims-technical-details.html#anker-7.

**Figure 6:**
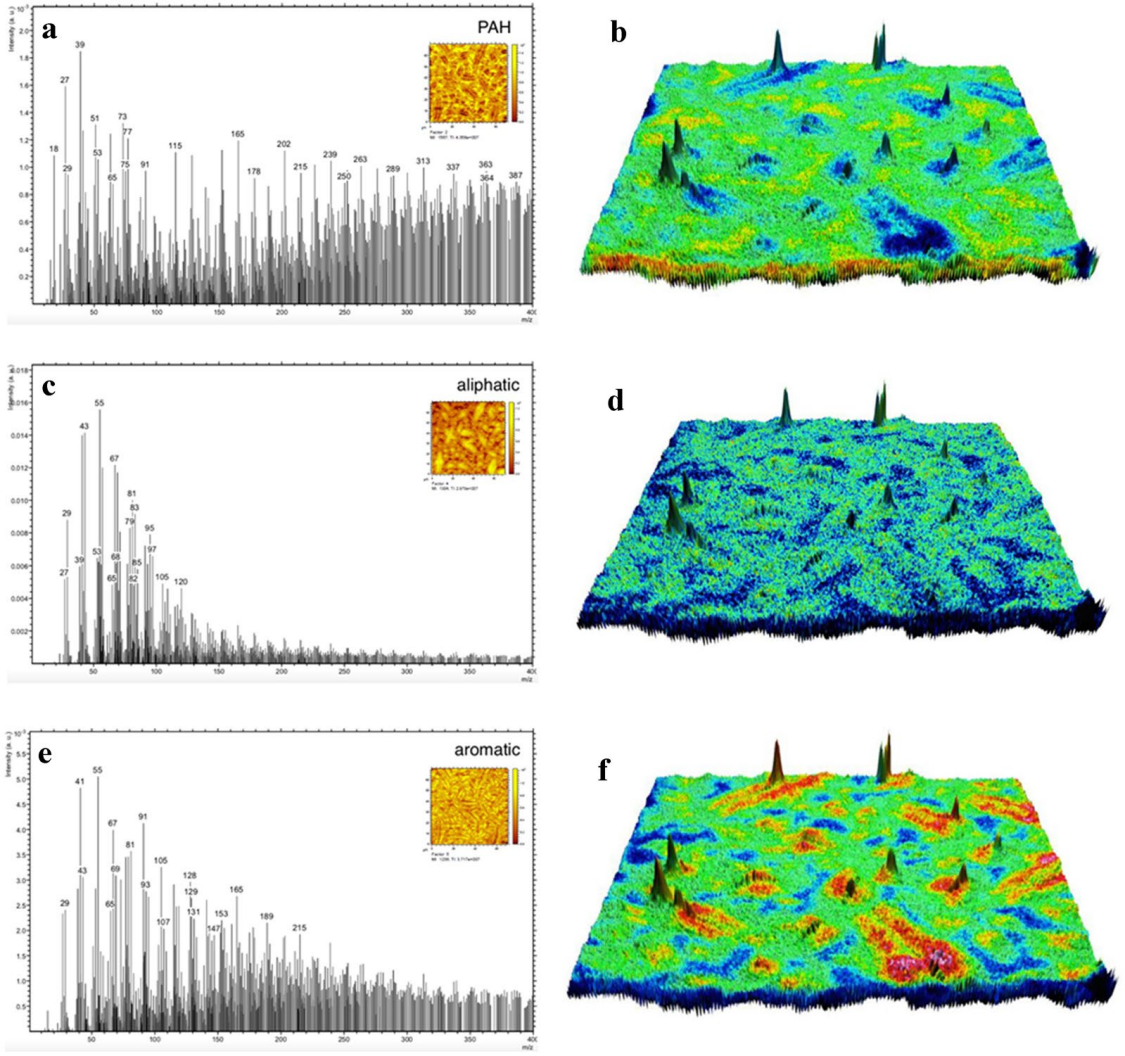
ToF–SIMS spectrum contributing to the ToF–SIMS image with resulting AFM-ToF–SIMS carpet plot images. (**a**) ToF–SIMS loadings spectrum correlated to polycyclic aromatic hydrocarbons which contributes to the SIMS score image on the top right corner, (**b**) AFM correlative ToF–SIMS carpet plot of high mass aromatic compounds. Here, red, green and blue areas indicate high, medium and low concentrations, respectively, (**c**) ToF–SIMS loadings spectrum correlated to aliphatic structures, which contributes to the SIMS score image on the top right corner, (**d**) AFM correlative ToF–SIMS carpet plot of combined aliphatic compounds, (**e**) ToF–SIMS loadings spectrum of combined aromatics which contributes to the SIMS score image on the top right corner, (**f**) AFM correlative ToF–SIMS carpet plot of combined aromatics. SIMS images—field of view: 54 × 54 µm^2^. Images created using SurfaceLab7 software. https://www.iontof.com/m6-tof-sims-technical-details.html#anker-7.

The correlation between AFM and ToF–SIMS images is presented in Figs. 5 and 6 (70 × 70 μm^2^ and a 54 × 54 μm^2^ field of view). Correlated key compounds^15^ were combined and their lateral distribution and intensities were visualized in Fig. 5 and in Supplementary Fig. 3. The contribution of each of these compound clusters was represented in an overlay image in Fig. 5 and in Supplementary Fig. 2 with a higher resolution. Characteristic aromatic compounds^15^ mainly found as (C_6_H_5_+, C_7_H_7_+, C_8_H_9_+, C_9_H_7_+, C_9_H_11_+, C_10_H_11_+ at *m/z* 77, 91, 105, 115, 119 and 131) are represented in Fig. 5g and make up mainly the catana (on the ripples) and peri phase, while the concentration in the catana phase is lower. The outer edge of the peri-phase shows a slightly lower number of aromatic compounds than the inner part of the peri phase. The lateral distribution of molecular compounds with a mass greater than 950 u, are typical indicators of asphaltenes with fused rings^48^ as well as high mass tail of aromatic compounds^49–53^, were found in higher concentration in the para phase and were less in the outer part of the peri phase and the catana phase. Resins corresponding to ions such as C_6_H_9_O_5_+ and C_7_H_11_O_5_+ were found on the outer edge of the peri phase. Aliphatic compounds (corresponding mainly to C_2_H_5_+, C_3_H_5_+, C_3_H_7_+, C_4_H_5_+, C_4_H_7_+, C_4_H_8_+, C_4_H_9_+, C_5_H_7_+, C_5_H_8_+, C_5_H_9_+, C_5_H_11_+, C_6_H_7_+, C_6_H_9_+, C_6_H_11_+, C_6_H_13_+, C_7_H_11_+, C_7_H_13_+) were detected in the peri phase with the highest concentration, a slightly lower concentration at the outer edge of the peri phase, and rather low concentration in the para phase. Overall, a spatial differentiation of various compounds was found between the phases. Aromatic compounds and aliphatic compounds occur in high concentrations in the catana and peri phase. Resin compounds are present on the outer edge of the peri phase, and asphaltenes contributed to the para phase.

Correlative AFM and ToF–SIMS were displayed in overlay images (carpet plots), where distinct areas displayed special chemical characteristics, and the well-known catana phase surrounded with peri and para phase are observed. Rather than sharply separated distinct domains of saturates, aromatics, asphaltenes, and resins the microstructure exhibits a more dispersed phase with varying concentration differences in and around the core of the microstructure. The loading plots shown in Fig. 6 and Supplementary Fig. 1 represent the chemical composition and its contribution to the corresponding score images on the top right side of the spectra. The plot in Fig. 6a,b clearly illustrates that the masses above 200 *m/z* and key compounds related to polycyclic aromatic hydrocarbons make a substantial contribution to the para phase (yellow areas in score image), whereas Fig. 6c,d shows that the majority of the contribution to the catana and peri phase originate from below 120 *m/z* and are of aliphatic origin. Masses over 950 *m/z* could be found in the para phase (Fig. 5e) likewise in relatively high concentration and decreasing towards the centre of the microstructure. Figure 6c shows the contribution of the aliphatic compounds mainly to the catana and peri phase. Figure 6e,f shows the aromatic compounds in a relatively higher concentration in the catana and peri- phase. The aromatics are found in a relatively high concentration in the inner catana phase (Fig. 6e,f), medium concentration in the outer edge of the peri phase and low concentration in the para phase.

Baheri et al. studied the temperature dependency of the microstructure formation with AFM-IR, where a coarsening of the para domain was observed^54^. Sulfoxides were found with a higher concentration in the para phase confirming our findings. We have conducted additional mapping for further information regarding the sulfoxide distribution. This visualization clarifies that the sulfoxide bands also occur at the outer edges and in the centres of the peri phases. Furthermore, we have found that the carbonyl groups occur mainly in the para phase. Lu et al. observed the same chemical contrast of the surface where two phases are visible—namely the aromatics (para phase) and aliphatic structures with a high degree of saturation (catana and peri phase)^15,18^. Our study supports these findings and reveals additionally the chemical contrast of the catana and the peri phase, which has now become possible with a much higher resolution of AFM-IR and AFM correlative ToF–SIMS. Furthermore, key compounds that are attributed to the resins are found in the outer part of the peri phase. With sulfoxides and carbonyl bands mainly occurring in the para phase we suggest that the para phase is more susceptible to oxidation. With the high concentration of asphaltene and resin structures in the para and the outer part of the peri phase identified with ToF–SIMS our findings confirm the consistency of our results since asphaltene and resin structures are highly susceptible to oxidation due to their incorporated heteroatoms. Fluorescence Microscopy delivers additional insight into the complex microstructure of bitumen and resolves the formerly observed ellipsoid structures and for the very first time resolves the fluorescing parts of the catana and peri phases^34^. It shows that the distribution of fluorescing compounds (aromatics and resins) in bee-structures are rather diffuse and fluorescence occurs only on the ripples of the catana phase while it is diffuse on the peri phase confirming our ToF–SIMS results.

## Discussion

In order to understand the atmospheric influence on the material’s surface, it is essential to investigate the surface composition of asphalt bitumen. In conclusion, AFM-IR, ToF–SIMS and fluorescence microscopy are powerful tools to investigate the multiphasic nature of asphalt bitumen surface. Here we have for the first-time presented complementary studies on the submicron and nanoscale, clearly demonstrating that the surface has a complex structural make up, consisting of elongated catana domains, surrounded by the para and peri phase exhibiting chemical differences. The three different phases (catana, peri and para phase) were identified with AFM-IR, ToF–SIMS and Fluorescence microscopy. The chemical heterogeneity is thought to cause self-organizing effects of the molecules due to their differences in polarity and intermolecular interactions such as π–π stacking. Fluorescence Microscopy observations identified aromatics and resins situated on the elongated catana domains with high concentration on the ripples and low concentration on the valleys of the catana phase which is in accordance with the data obtained from AFM–ToF–SIMS analysis. High mass aromatic compounds were found mainly in the para phase, while aliphatic compounds formed the catana phase. The lateral distribution of functional groups was found along the surface at a nanoscale clearly showing high concentration of sulfoxide and carbonyl mainly in the para phase and the outer part of the peri phase, confirming the highly reactive nature of asphaltenes and resins due to their high content in heteroatoms and eventually metals (for asphaltenes) increasing their reactivity with reactive oxygen compounds. Phase separation of the surface is clearly paving the way to a selective oxidation due to varying susceptibility of the surface domains to atmospheric oxidation and as a result of chemical heterogeneity changing its physicochemical properties on certain domains.

Fluorescence Microscopy, AFM correlative ToF–SIMS and AFM-IR observations of the bitumen surface as an intercomparing method have been used to develop a model of the chemical composition of the surface microstructure. These experiments deliver microscopic and spectroscopic information of the surface of bitumen where each catana, peri and para phase could be distinguished and resolved at a resolution which has not been previously achieved. It is important to emphasize that the complex chemistry of bitumen, which consists of thousands of different molecules, contributes to the complexity of the microstructure chemistry while the combination of complementary methods with high spatial resolution is key to obtaining the required compositional information.

## Materials and methods

### Preparation of experimental bitumen

Oxidized asphalt with bitumen specifications 90/10 (unmodified and no further addition of wax) according to Austrian Standard (ÖNORM B 3613, 2018) was selected, due to its widely use in a conventional asphalt. The bitumen was heated to 120 °C and then spin coated at a constant speed of 5000 r.p.m. for 2 min on an Au-coated Silicon Wafer with a film thickness of 400 nm (measured with the vertical profiling mode of Witec Alpha RSA + instrument). A spin coater from Spin Coater from Spin Coater Systems (SCS) model P6700 was used. The sample was tempered at 90 °C for 30 min and then cooled down to 23 °C overnight. This bitumen has a penetration grade of 14 mm/10 with a softening point from ring and ball test of 90.5 °C.

### Fluorescence microscopy

Taking advantage of the auto-fluorescence of aromatic, resin compounds which show auto fluorescence due to their molecular composition with conjugated double bonds a differentiation/intrinsic selectivity to the saturates phase has been made. Asphaltenes are capable of auto-fluorescence, however, they do not exhibit fluorescence in the selected excitation and emission range and therefore are not captured in this particular fluorescence image. Fluorescence images are recorded using a Nikon Eclipse Ci-L instrument. The setup is equipped with a Plan Fluor 100 × objective with a numerical aperture of 0.9. An epifluorescence unit with an excitation filter at 465–495 nm, a dichroic mirror at 505 nm and an emission filter at 515–555 nm are used. A 100 W metal-halide light source and a Nikon color-digital camera DS-Fi3 captured images using a Nikon software NIS Elements BR with an illumination time set to 700 ms.

### AFM-IR measurements

The localized nanoscale midIR spectra and images are carried out using the commercial Bruker NanoIR3 AFM-IR instrument (Bruker/Anasys Instruments, Santa Barbara (CA), USA). The AFM-IR technique^55^ is accomplished by coupling a pulsed tunable IR source with an AFM.

The IR source is focused on the area around the tip on the sample and causes local photothermal expansion, when the IR radiation is absorbed by the material. The periodic expansion generated by the laser pulses is picked up the by AFM tip and results in a specific oscillation of the AFM cantilever, whereby the amplitude of the recorded oscillation is proportional to the increase of the local temperature, which is directly proportional to the local absorption coefficient of IR active compounds. The IR source used in the present study is a tunable 4-chip midIR QCL Laser system, which covers the 912–1958 cm^−1^ range of the mid-IR region.

IR absorption maps at various wavenumbers of interest as well as nanoscale local IR spectra and the topography images were all acquired via the Tapping AFM-IR Mode due to the viscoelastic nature of bitumen^56,57^. Tapping AFM-IR Mode is based on non-linear mixing phenomena in a heterodyne force microscopy approach. The pulse rate is chosen as difference frequency between two cantilever eigenmodes, where one eigenmode (here: the first eigenmode) is used for tapping topography feedback, and the other one (here: the second eigenmode) is used for IR detection. For AFM-IR measurement a gold coated probe was used (Model: PR-EX-TnIR-A-10, Anasys Instruments) with a resonance frequency of 300 kHz, a spring constant of 40 N/m, and a second eigenmode of ~ 1800 kHz.

### AFM-ToF–SIMS

Positive ion data are recorded with the TOF–SIMS M6 Plus (IONTOF GmbH, Münster Germany) instrument which is optimized for a high lateral resolution (< 50 nm) with in-situ SPM capabilities with a mass resolution of > 240,000 and < 1 ppm mass accuracy. AFM Images are recorded in contact mode at − 150 °C. After the SPM measurement, ToF–SIMS measurements are performed, where a focused pulsed primary ion beam hits the solid sample surface, resulting in the emission of atomic and molecular ions (secondary ions) from the first nanolayers of the surface. The differentiation of the emitted ions is using the difference in time of flight of each ion, which is proportional to the square root of molecular weight. Bi^3+^ is used as a primary ion source. The primary ion energy of 30 keV and an analysis current of 0.05 pA are selected. Analysis area of 54 × 54 µm^2^ with a raster size of 512 × 512 Pixels are selected respectively. The sample is cooled during the analysis and sample transfer at − 150 °C ensuring stability of microstructures in UHV conditions during the entire procedure. Multivariate statistical analysis methods (MVSA) are applied to obtained data sets to reduce the degree of complexity and to determine the major chemical components and their contribution to the composition of the surface. Multivariate Curve Resolution (MCR) Analysis were applied and score images are generated using SurfaceLab7 software (IONTOF GmbH, Münster Germany), which revealed specific domains clearly representing the surface microstructures. The combination of SPM and ToF–SIMS in situ given with the lateral resolution of below 50 nm allows a true overlap of both chemical and morphological information resulting in carpet plots.

## Supplementary Information

Supplementary Information.

## Data Availability

The data that support the findings of this study are available from the authors on reasonable request (see author contributions for specific data sets).
