# Author Correction: High resolution nanoscale chemical analysis of bitumen surface microstructures

**DOI:** 10.1038/s41598-021-96410-8

**Published:** 2021-08-19

**Authors:** Ayse N. Koyun, Julia Zakel, Sven Kayser, Hartmut Stadler, Frank N. Keutsch, Hinrich Grothe

**Affiliations:** 1 Christian Doppler Laboratory for Chemo‑Mechanical Analysis of Bituminous Materials, Institute of Materials Chemistry, TU Wien, Getreidemarkt 9/BC, 1060 Vienna, Austria; 2 IONTOF GmbH, Heisenbergstrasse 15, 48149 Münster, Germany; 3 Bruker Nano-Surfaces Division, Östliche Rheinbrückenstrasse 49, 76187 Karlsruhe, Germany; 4 John A. Paulson School of Engineering and Applied Sciences, Harvard University, Cambridge, MA 02138 USA

Correction to: *Scientifc Reports* 10.1038/s41598-021-92835-3, published online 30 June 2021

The Funding section in the original version of this Article was omitted. The Funding section now reads:

“The authors acknowledge TU Wien Bibliothek for financial support through its Open Access Funding Programme.”

The original Article has been corrected.

