# Supplementary material for: High resolution nanoscale chemical analysis of bitumen surface microstructures
Source: Sci Rep. 2021 Jun 30;11:13554. doi: 10.1038/s41598-021-92835-3 (PMC8245519; doi:10.1038/s41598-021-92835-3)
Supplement: Supplementary file 1 — Supplementary Information. [file 41598_2021_92835_MOESM1_ESM.pdf]

Supplementary Materials for

High resolution nanoscale chemical analysis of bitumen surface microstructures

Ayse N. Koyun*, Julia Zakel, Sven Kayser, Hartmut Stadler, Frank N. Keutsch, Hinrich Grothe*

*Corresponding author. Email: akoyun@seas.harvard.edu and hinrich.grothe@tuwien.ac.at

This supplemental material presents the reproduction of atomic force microscopy (AFM) coupled time of flight secondary ion mass spectrometry (ToF-SIMS) on an additional sample spot with an extended mass range of 0-2000 m/z.

This PDF file includes:

Supplementary Text
Figs. S1 to S3

Supplementary Text

<u>ToF-SIMS</u>

Extended ToF-SIMS data with the total range from 0-2000 m/z is demonstrated in Figure S1. Reproducibility of the obtained chemical pattern is given on an additional sample spot and is represented in Figure S2 and S3.

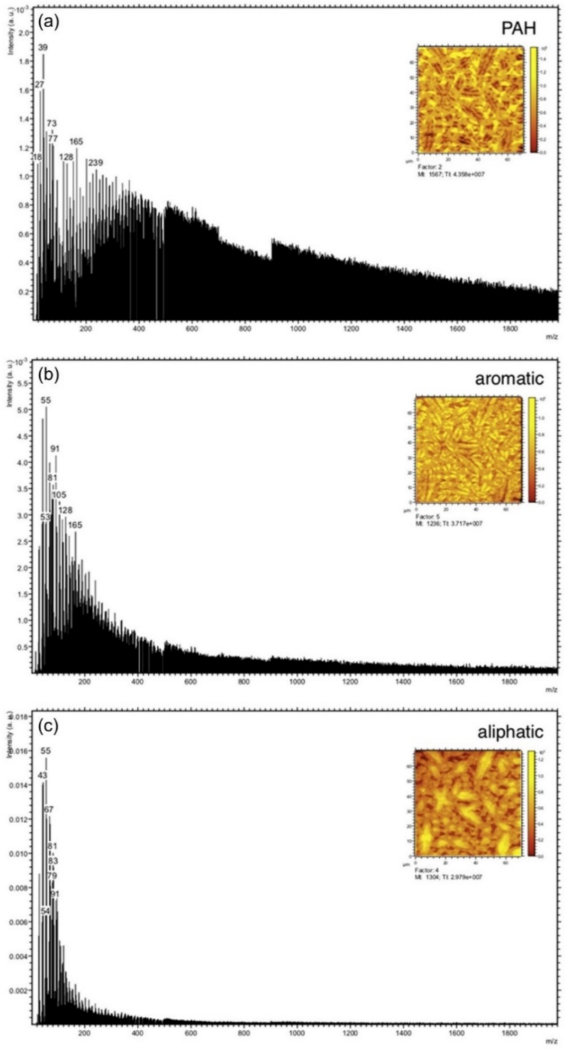

Fig. S1.

ToF-SIMS data including the range above 400 m/z. The total range of ToF-SIMS data from 0-2000 m/z demonstrate the contribution of high mass compounds to the SIMS image on the top right corner for a) polycyclic aromatic compounds b) aromatic compounds c) aliphatic compounds.

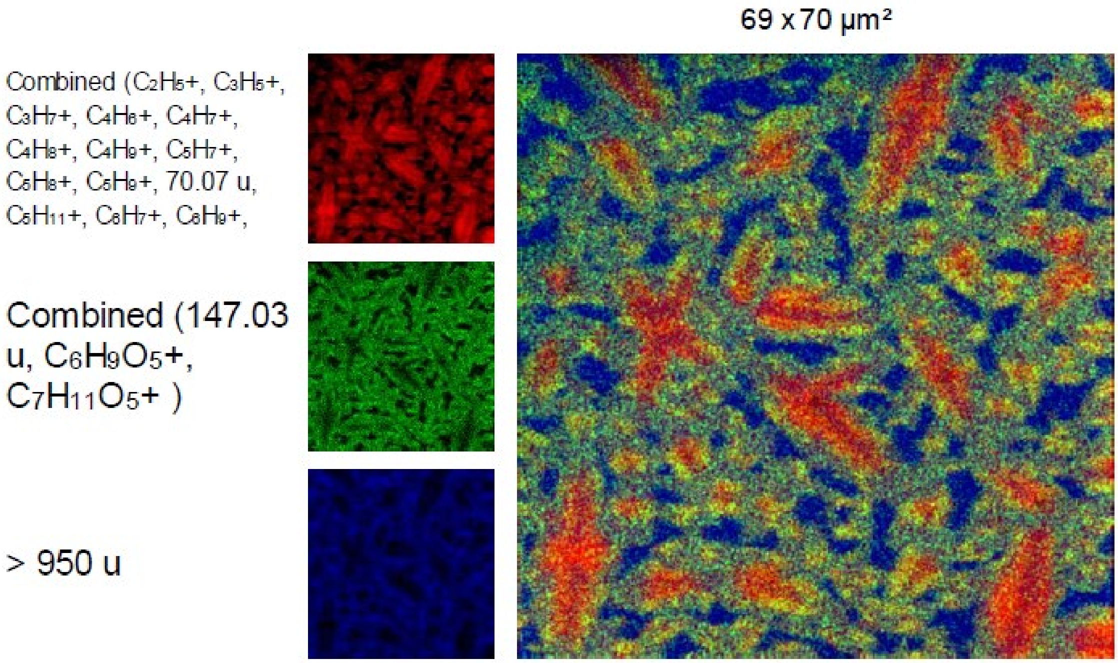

Fig. S2.

ToF-SIMS overlay images. Overlay image considering the contributions of aliphatic (dyed in red), resin (dyed in green) and asphaltene (high mass > 950 u) (dyed in blue) compounds.

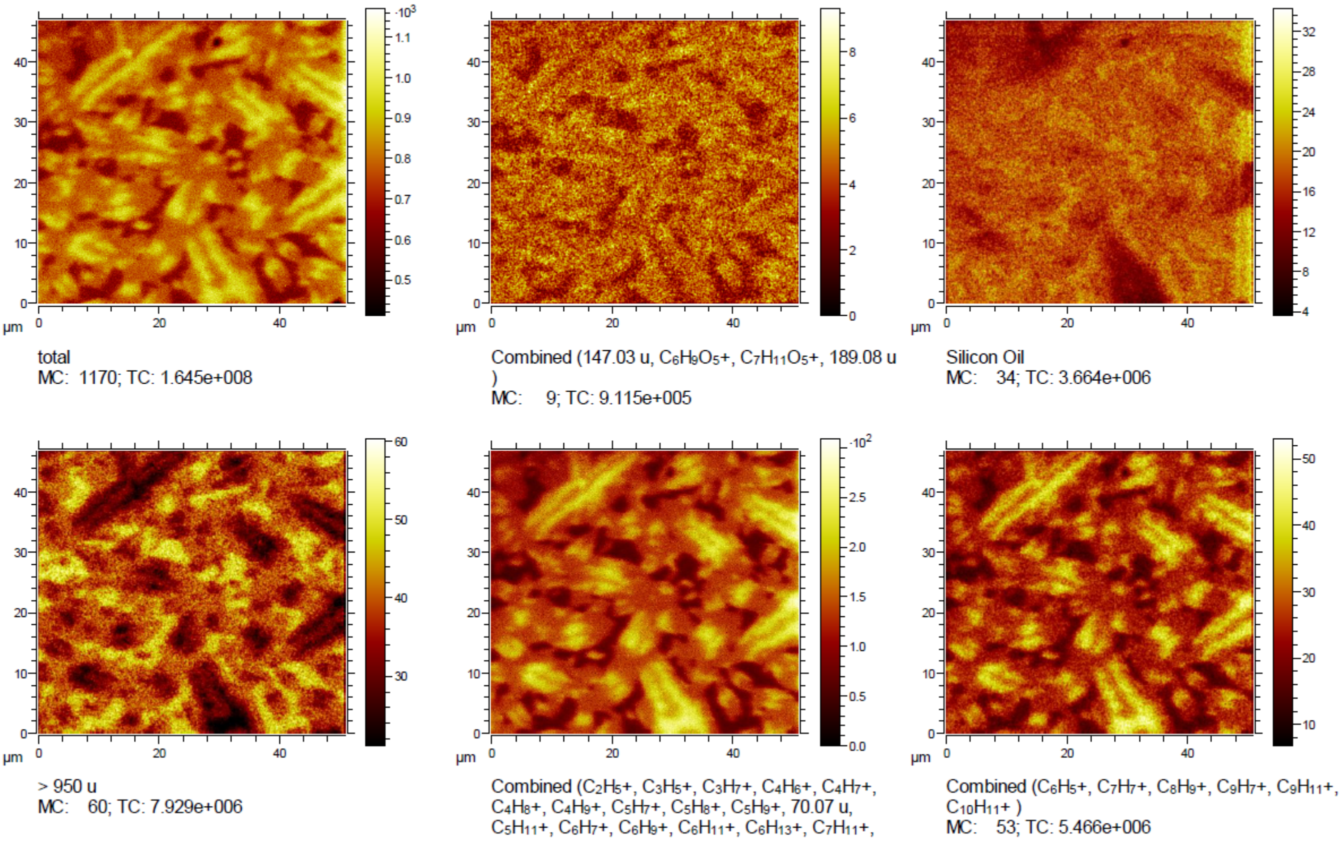

Fig. S3.

Positive polarity ToF-SIMS images of the bitumen surface. Field of view: 54 x 54 μm2. Correlated key compounds contributing to different phases of surface microstructures.
